# Gsy, a novel glucansucrase from *Leuconostoc mesenteroides*, mediates the formation of cell aggregates in response to oxidative stress

**DOI:** 10.1038/srep38122

**Published:** 2016-12-07

**Authors:** Minghui Yan, Jin Han, Xiaofen Xu, Lianliang Liu, Caixia Gao, Huajun Zheng, Yunxia Chen, Yimin Tao, Hu Zhou, Yunfei Li, Zhengjun Wu

**Affiliations:** 1 State Key Laboratory of Dairy Biotechnology, Institute of Bright Dairy & Food Co., Ltd., 1518 West Jiangchang Road, Shanghai 200436, PR China; 2 Department of Food Science and Technology, School of Agriculture and Biology, Shanghai Jiao Tong University, Shanghai 200240, PR China; 3 School of Food Science and Technology, Jiangnan University, 1800 Lihu Rd,Wuxi,214122, PR China; 4 Shanghai-MOST Key Laboratory of Health and Disease Genomics, Chinese National Human Genome Center at Shanghai, Shanghai Zhang Jiang Hi-TechPark, 250 Bi-Bo Road, Shanghai 201203PR China; 5 Department of Analytical Chemistry, Shanghai Institute of Materia Medica, Chinese Academy of Sciences, Shanghai, 201203, PR China; 6 School of Marine Sciences, Key Laboratory of Applied Marine Biotechnology (Ministry of Education), Ningbo University, Ningbo, 315211, PR China

## Abstract

*Leuconostoc mesenteroides* is a member of lactic acid bacteria (LAB) with wide applications in the food and medical industries. Species in the genus *Leuconostoc* are catalase-negative and generally regarded as facultative anaerobic or aerotolerant organisms. Despite their extensive use in industry, certain issues concerning the aerobic life of *L. mesenteroides*, e.g., the mechanism involved in the tolerance to oxygen, remain to be addressed. In this manuscript, a survival strategy employed by *L. mesenteroides* BD3749 in response to oxidative stress was elucidated. BD3749 cells cultivated in medium with sucrose available synthesized large amounts of exopolysaccharides, mostly consisting of insoluble EPS. When BD3749 cells were challenged with oxidative stress, the amount of insoluble EPS was greatly enhanced. The synthesized EPSs reduced the accumulation of reactive oxygen species (ROS) in bacterial cells and improved their survival during chronic oxidative stress. Another study showed that Gsy, a novel glucansucrase in the GH70 family that is induced by sucrose and up-regulated following exposure to oxygen, was responsible for the synthesis of insoluble EPS. Gsy was subsequently demonstrated to play pivotal roles in the formation of aggregates to alleviate the detrimental effects on BD3749 cells exerted by oxygen.

Oxidative stress is a constant challenge for most life forms in nature, and it is predominantly derived from normal cell activities such as electron transport during cellular respiration. When molecular oxygen serves as an acceptor for one or two electrons, toxic superoxide or peroxide anions, respectively, are produced^1^,^2^,^3^. In some circumstances, oxidative stress may also arise from different stimuli in the environment, such as reactive oxygen species (ROS) and ionizing radiation^4^.

Reactive oxygen species, e.g., superoxide and hydrogen peroxide, are highly reactive radicals and thus pose threats to biomolecules such as proteins and nucleic acids^5^,^6^,^7^,^8^. In response to these oxidative stresses, most life forms have developed enzyme systems to scavenge ROS, among which are the most widely studied superoxide dismutase (SOD) and catalase. SOD, along with its substitute SOR (superoxide reductase) in anaerobes, provides an important defence against the toxicity of oxygen by transforming superoxide into the less toxic hydrogen peroxide. However, exceptions have been found in lactic acid bacteria. For example, *Lactobacillus plantarum* and related lactobacilli, which lack SOD/SOR, employ a manganese-dependent mechanism to prevent damage caused by reactive superoxides^9^,^10^,^11^,^12^,^13^. Furthermore, catalase and its substitute peroxidase in some anaerobes may provide their hosts with complementary defence mechanisms to scavenge peroxides, which may be a by-product of SOD in the cell, or a stimulus from the environment. Additionally, many aerotolerant bacteria have developed peroxidases such as thiol peroxidase and glutathione peroxidase to neutralize these lethal compounds in cells^14^.

SOD/SOR-negative LABs are mostly strict anaerobes or facultative anaerobes, and thus exposure to oxygen may be detrimental due to a failure to deal with ROS. *Leuconostoc* species are epiphytic and widely spread in natural environments. *L. mesenteroides* plays important roles in several industrial and food fields, such as the fermentation of kimchi in East Asia and sauerkraut in Europe. *L. mesenteroides* is generally regarded as a facultative or aero-tolerant anaerobe that requires complex growth factors and amino acids^15^,^16^,^17^,^18^. During growth in sucrose medium, *L. mesenteroides* produces large amounts of exopolysaccharides (EPS) using sucrose as substrate^19^. EPS synthesized by *L. mesenteroides* are mostly water-soluble dextran or levan, which have been applied in the pharmaceutical as well as the food industry. For example, dextrans with various molecular weights can be used commercially as an antithrombotic agent to reduce blood viscosity, or as a volume expander in hypovolemia. Additionally, EPS from some strains of *L. mesenteroides* contribute to the gelatinous texture of fermented milk products. Due to the important commercial value of dextran, the process of dextran synthesis has been extensively studied, and several dextransucrase-encoding genes have been cloned from different strains of *L. mesenteroides*^20^,^21^,^22^,^23^. Most strains of *L. mesenteroides* are able to produce water-soluble EPS, only a few strains have been reported to synthesize insoluble EPS^24^,^25^. Although rarely seen in *L. mesenteroides*, insoluble EPS are a common phenomenon in *Streptococcus mutans* and *Lactobacillus reuteri*, and play critical roles in biofilm formation, stress resistance and adhesion^26^,^27^,^28^,^29^. To date, relatively little is known about the biological effects of insoluble EPS on *L. mesenteroides*. Considering the wide application of *L. mesenteroides* in the food and medical industry, it is thus necessary to elucidate the role of insoluble EPS in the life cycle of *L. mesenteroides*, especially in terms of its relationship with oxygen.

In this manuscript, we investigated BD3749, a strain of *L. mesenteroides* that is capable of synthesizing large amount of both soluble and insoluble EPSs. The biochemical studies suggested that the glucansucrase, Gsy, mediated the up-regulation of EPS synthesis and subsequent aggregation of cells in response to oxidative stress. The role of a novel glucansucrase and its insoluble EPS product in the adaptation of *L. mesenteroides* to the aerobic environment was also demonstrated.

## Materials and Methods

### Strains and culture conditionhs

*L. mesenteroides* BD3749 (=CGMCC10064) was provided by the State Key Laboratory of Dairy Biotechnology, Bright Dairy & Food Co., Ltd., Shanghai, China. The bacterial strain was routinely streaked on MRS agar (Merck, Germany) supplemented with 5% sucrose and incubated at 30 °C under anaerobic conditions^30^. For EPS production, an overnight culture of BD3749 was diluted 1:100 into fresh liquid medium adapted from Liu *et al*.^31^ containing the following (g/L): sucrose, 200; tryptone, 10; yeast extract,5; K_2_HPO_4_, 5; and CaCl_2_, 0.34, with the pH adjusted to 6.5. For induction of cell aggregation and expression of glucansucrase, the concentration of sucrose in the medium described above were lowered to 20 g/L according to Tsuchiya *et al*.^32^, and CaCl_2_ was removed to restrict the synthesis of exopolysaccharides, which may interfere with the precipitation of proteins by (NH_4_)_2_SO_4_ in the cultivated broth.

Anaerobic cultivation was carried out in a Whitley A35 anaerobic workstation (don Whitley scientific, UK) filled with a mixture gas of N_2_:H_2_:CO_2_ = 85:10:5 (v/v). Aerobic cultivation on agar plates was conducted by exposing the plates to air or in flasks containing liquid medium on a rotatory shaker at 180 rpm. To mimic oxidative stress, H_2_O_2_ was added to flasks containing liquid medium at a final concentrations of 50 or 500 μM via 0.22-μm membrane filtration (at a concentration higher than 2 mM, H_2_O_2_ inhibits the growth of BD3749), and then the cultivation was performed in the anaerobic workstation described above.

### Microscopy

A logarithmic phase culture of strain BD3749 in liquid medium containing 20 g/L sucrose cultivated under aerobic or anaerobic condition was allowed to set at room temperature for 5 minutes for natural sedimentation. The precipitate and supernatant were sampled and observed with a Carl Zeiss AxioPlan 2 Imaging system, and the cells were designated as aggregated and planktonic cells, respectively.

### Quantification of EPS

To quantify insoluble EPS, 200 mL cultivated broth was centrifuged at 10,000 × g for 10 min at 4 °C after the broth was diluted by the addition of four volumes of distilled water. The precipitate was washed 3 times with deionized water, freeze-dried (Freezone12, Labconco, USA) and weighed. The weight of the precipitate was normalized by subtracting the weight of the control group, which was cultivated with glucose as the sole carbon source, indicating the final amount of insoluble EPS. Soluble EPS was measured as described in a previous study^30^. Briefly, four volumes of pre-chilled ethanol was added to the supernatant, and the mixture was stored overnight at 4^ ^°C. The precipitate was collected by centrifugation at 15,000 × g at 4 °C for 30 min, re-dissolved and dialysed in a membrane tube with a MWCO of 14000 Da against deionized water at 4 °C for 24 h. The yield of soluble exopolysaccharide was determined by weighing the retentate after freeze-drying. Unless specifically mentioned, the amount of EPS was measured in culture broth after 48 h of cultivation.

### ROS measurement

The ROS level was determined using 2, 7-dichlorodihydrofluorescein diacetate (H_2_DCF-DA) (Beyotime Institute of Biotechnology, Haimen, China) according to the instructions provided by the manufacturer. Approximately 1 × 10^6^ cells were collected and washed with PBS and then treated with 10 μM H_2_DCF-DA dissolved in PBS at 37 °C under anaerobic conditions for 20 min. After removal of H_2_DCF-DA and three washes with PBS, the fluorescence intensity was monitored at an excitation wavelength of 488 nm and an emission wavelength of 525 nm.

To measure the ROS level in cells within the aggregates, the aggregates were first treated with 0.5 mol/L NaOH for 5 min to dissolve the insoluble EPS with slight shaking. The cells were collected by centrifugation and treated as described above. The intensity of DCF fluorescence was quantified using a micro plate assay with SpectraMax M5 (Molecular Devices, USA). An equivalent volume of each sample was sonicated and subjected to quantification of the total protein using the Bradford method. The fluorescence intensity was normalized to the total protein content, and the relative amount of ROS is depicted as the DCF fluorescence intensity per mg total protein.

### Protein extraction, SDS-PAGE, and western blot analysis

To isolate glucansucrase from the supernatant, (NH_4_)_2_SO_4_ was added to the final saturation of 50%, and the mixture was centrifuged at 15,000 × g at 4 ^o^C for 15 min. The precipitate was then dissolved in pure water and quantified using the Bradford method. Equivalent amount of total protein was separated by 8% SDS–PAGE and stained with Coomassie brilliant blue G250 or transferred to polyvinylidene difluoride (PVDF) membranes (Bio-Rad). After blocking in 5% non-fat milk, the membranes were probed with specific antibodies, followed by visualization using the ECL detection system (Pierce, Thermo).

Antibodies were raised against chemically synthesized peptides. Briefly, the peptides were synthesized (GL Biochem, Ltd.) and coupled to KLH through the bi-functional coupling agent sulfo-smcc (Thermo). The coupled antigen was then employed to immunize two New Zealand rabbits. After titre determination with ELISA, the serum was affinity-purified using a thiol-coupled column (Thermo). The specificity of the purified antibody was then confirmed by western blot analysis, and the antibody with the highest specificity was chosen for further analysis of the indicated protein. Three antibodies each were raised against BD3749_1322 (renamed Gsy in this work) and BD3749_1323, and the one with the best specificity and titre was chosen for further analysis. The results obtained using the antibody raised against peptides N-KYVNDSETSWSD-C (anti-1322) and N-DTNHNATTQAL-C (anti-1323) are shown.

### *In situ* polymer synthesis

For *in situ* detection of glucansucrase activity, after electrophoresis, the SDS-PAGE gel was incubated in 50 mM sodium acetate buffer containing 50 g/L sucrose with a pH value of 5.6, as described in previous studies^33^. Briefly, the gel was first washed three times with 50 mM sodium acetate buffer (pH 5.4) containing 2 mM CaCl_2_ and 0.1% (vol/vol) Triton X-100 at room temperature to eliminate the SDS. It was then incubated in the same buffer supplemented with 50 g/L sucrose at room temperature for 24 h, and the active bands were detected by the appearance of glucan polymer as described by Miller and Robyt^33^,^34^.

### In-gel Digestion and MS analysis

The differentially expressed protein bands were excised from the CBB-stained SDS-PAGE gel and cut into small pieces in a 1.5-ml micro tube. To remove the Coomassie Brilliant Blue dye, the chopped gels were washed three times with 50 mM NH_4_HCO_3_ in 30% acetonitrile by shaking at room temperature for 20 min. The gels were then further incubated with 300 μL of acetonitrile at room temperature for 10 min. After removing the acetonitrile, the gel slices were reduced by incubation with 20 mM dithiothreitol at 56 ^o^C for 30 min and then alkylated by incubation with 100 mM iodoacetamide in the dark at room temperature for 20 min. The gels were dried and trypsin-digested following the manufacturer’s protocol. Peptides were extracted twice with 85% acetonitrile and 0.1% trifluoroacetic acid. After removing the pieces of gels, the remaining solution was concentrated using a SpeedVac concentrator. The peptides were then re-dissolved in 30 μL of 0.1% formic acid for LC-MS/MS mass spectrometry analysis (LTQ Orbitrap Elite, Thermo Scientific). The fragment ions observed in the mass spectra were analysed using Thermo Proteome Discoverer 1.3 (Version 1.3.0.339).

### Quantitative reverse-transcription PCR

Cell cultures were collected and treated with RNA protection reagent (Qiagen). Total RNA extraction and transcription analysis was performed as previously described^35^. Briefly, RNA was extracted and purified using RNeasy Mini kits (Qiagen) and digested with RNase-free DNase I. The concentration of RNA was measured using a Nanodrop ND 1000 spectrophotometer (Thermo Fisher Scientific). Agarose electrophoresis was then used to determine the integrity and quality of the RNA. For Quantitative reverse-transcription PCR analysis, gene-specific primers (Table 1) were used, and first-strand cDNAs were synthesized using the FastQuant RT kit with gDNase (Tiangen) according to the manufacturer’s instructions (a none RT control was done as described previously^35^ to ensure to absence of gDNA). Each PCR reagent (25 μL) contained SuperReal premix Plus (Tiangen), cDNA samples and forward and reverse gene-specific primers (10 μM each). The thermo-cycling conditions were 95 °C for 30 s, and 40 cycles at 95 °C for 30 s and 60 °C for 30 s. The amplification specificity was assessed using melting curve analysis. Differential gene expression levels were normalized to the levels of 16 S rRNA gene transcripts. The anoxic conditions were used as reference conditions and the relative transcription level under oxic conditions was determined by fold to that of the anoxic reference.

### Survival experiments in sucrose medium free of antioxidants

An overnight culture of BD3749 in MRS broth was inoculated into 30 mL TYC medium^36^ or TYC-Glc medium (in which sucrose was substituted with an equal amount of glucose) at a ratio of 3% (v/v) and incubated anaerobically at 30 °C for 48 h. The cultures were then placed in an incubator at 30 °C and maintained for 5 days, during which the pH value was monitored and the number of viable cells (CFU/mL) was determined everyday by diluting the culture aliquots and plating on MRS agar.

### Nucleotide sequences

The 16 S rRNA gene sequence has been submitted to GenBank under accession number KU207096. The nucleotide sequence of *gsy* (BD3749_1322) and the other three putative GS-encoding genes (BD3749_1323, 1645 and 1650) have been submitted, and the accession numbers are KU306931, KU306932, KU306933 and KU306934, respectively. The genome sequence of BD3749 has been submitted under accession number CP014610.1.

## Results

### BD3749 forms aggregates in response to oxidative stress

BD3749 was originally isolated from a traditional fermented food in the southeast of China and identified as *L. mesenteroides* based on its phenotypic and genotypic properties. When cultivated on agar plates containing sucrose, BD3749 formed large, centrally convex colonies with a wrinkled edge.

During cultivation in sucrose-containing broth under aerobic conditions with stirring, the cells of BD3749 formed obvious aggregates. As EPSs had been shown to participate in biofilm formation^37^,^38^ and cell adhesion^39^, we speculated that EPS synthesized by strain BD3749 might play critical roles in aggregate formation. This speculation was confirmed by the observation that cells cultivated in glucose medium failed to aggregate under the same conditions, indicating that the formation of aggregates was dependent on the EPS synthesized from sucrose. Furthermore, the role of aeration in the formation of aggregates was explored. The formation of aggregates was aeration-dependent, as cells grown in the same broth did not form aggregates without aeration (data not shown).

**Figure 1:**
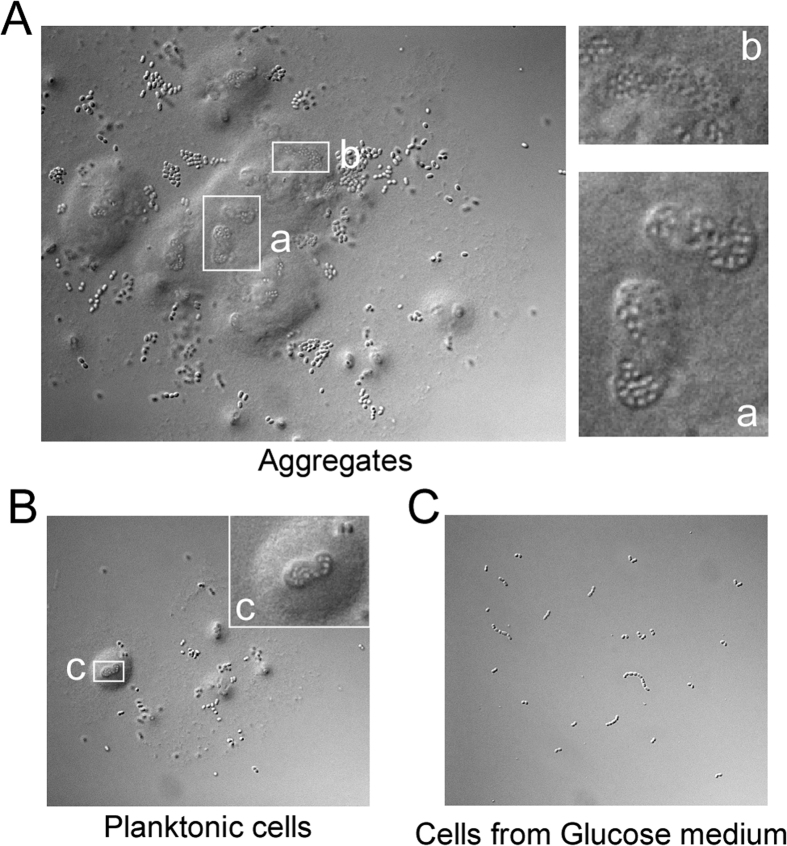
Sucrose-dependent aggregation of BD3749 in response to oxygen. (**A,B,C**) An overnight culture of BD3749 was diluted 1:100 in fresh medium containing 2% glucose or sucrose and incubated aerobically at 180 rpm and 30 °C. The aggregation characteristics of logarithmic-phase culture in glucose or sucrose was investigated and photographed. Shown are the microscopic images of BD3749 in aggregated (**A**) and planktonic (**B**) cells grown in sucrose medium. Cell morphology of BD3749 grown in glucose medium is shown in (**C**). All microscopic images were recorded with an AxioPlan 2 Imaging system by Carl Zeiss. The boxed areas were enlarged on the right.

Microscopic observation indicated that the aggregates mainly consisted of BD3749 cells covered by a large amount of insoluble substances (Fig. 1A). In contrast, such a complex was not observed in BD3749 cells grown in broth containing glucose (Fig. 1C), suggesting that this complex was associated with EPS produced from sucrose. Interestingly, a similar complex was observed in the planktonic portion, although on a smaller scale, which was considered to be an earlier form of aggregates. With a longer exposure to oxygen, these smaller-scale complexes increased in size and formed new aggregates (Fig. 1B).

### Exposure to oxygen leads to an accumulation of ROS in BD3749 cells

*L. mesenteroides* is generally catalase-negative, and exposure to oxygen may be harmful to bacterial cells due to the failure to scavenge reactive oxygen species (ROS). Although BD3749 showed no obvious growth on TYC plates under aerobic condition, following supplementation with Mn^2+^ and vitamin C, BD3749 displayed vigorous growth (Fig. S1), suggesting that the growth failure on TYC plates incubated aerobically may have been due to oxidative stress imposed by oxygen.

**Figure 2:**
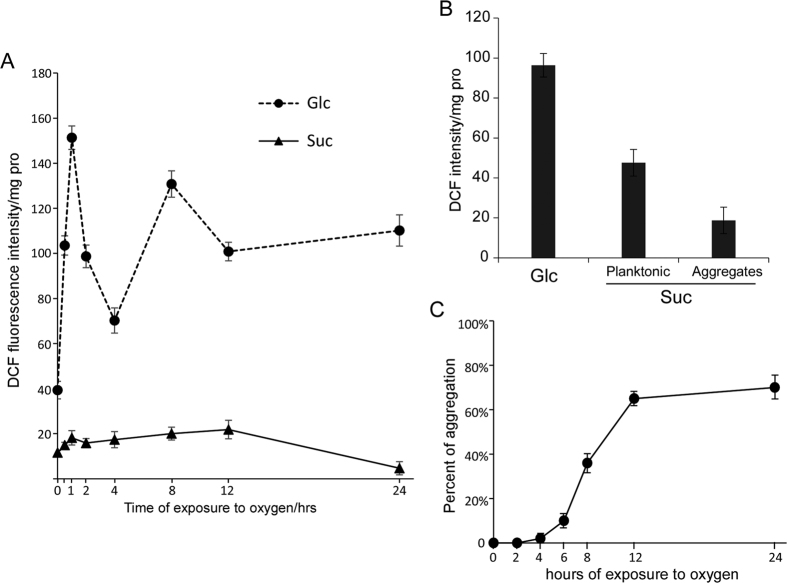
ROS accumulation in BD3749 exposed to oxygen. (**A**) Relative ROS accumulation in BD3749 cells grown aerobically in medium containing glucose or sucrose as a sole carbon source, as described in Materials and Methods. The amount of ROS was calculated by the intensity of the DCF fluorescence per mg total protein of the cell lysate. The relative ROS level of the BD3749 culture in glucose (Circle) and sucrose (Triangle) are shown. Experiments were performed in triplicate, and the average intensity and standard deviations are shown. (**B**) Relative ROS level in aggregated and planktonic cells of BD3749. A logarithmic culture was set on the table for 2 min for natural sedimentation of the aggregates. Aggregated and planktonic cells were collected independently, and the ROS level was monitored as described in Materials and Methods. (**C**) The percent aggregation was monitored for 24 hours of aerobic growth. Aggregated cells were collected as described above, and planktonic cells were harvested by centrifugation at 8000 rpm for 5 minutes. The collected cells were then resuspended in PBS to a volume equivalent to that of the culture after 3 washes with 0.5 M NaOH before sonication. The protein content of sonicated cell lysate was then quantified using the Bradford method. The percent of aggregation was calculated by protein content of aggregated cells divided by that of aggregated cells plus planktonic cells. All the experiments were performed in triplicate, and the standard deviations are shown in the graphs.

To further explore the oxidative stress challenge exerted by environmental oxygen to BD3749, the ROS level inside bacterial cells was examined. As shown in Fig. 2A, the relative level of ROS peaked in BD3749 cells after two hours of exposure to oxygen. Thereafter, the ROS level decreased and was restored to normal levels after approximately four hours of exposure to oxygen. However, persistent exposure to oxidative stress subsequently resulted in an accumulation of ROS unless the bacterial cells could form an alternative protective barrier. Compared with cells grown in sucrose, those grown in glucose encountered another burst of reactive oxygen species after exposure to oxygen for more than 6 h, while the ROS in the former were maintained at a relatively low level (Fig. 2A), which can be reasonably attributed to the synthesis of EPS from sucrose by the bacterial cells. Thus, it can be concluded that EPS provided BD3749 with protection against oxidative stress.

### Aggregated cells are less challenged by oxidative stress

Since the cells of strain BD3736 grown in sucrose medium exposed to oxygen synthesized EPS and thus formed aggregates with much of the bacterial cells encapsulated, the aggregates might play critical roles in protecting cells against oxidative stress. Therefore, the ROS levels in the aggregated and planktonic cells were carefully examined (Fig. 2B). Compared with BD3749 cells cultivated in glucose medium, much lower ROS levels were detected in aggregated cells in sucrose medium. Additionally, the ROS levels in planktonic cells in sucrose medium were also reduced, although much higher than those in aggregated cells. These results provided strong evidence that the formation of aggregates could alleviate oxidative stress to BD3749 cells and that the smaller cell-EPS complexes in the suspension portion were in the process of aggregating. The dynamic process of aggregation, as shown in Fig. 2C, provided further evidence that planktonic cells gradually formed aggregates with persistent exposure to oxygen.

### Analysis of glucansucrases in BD3749

**Figure 3:**
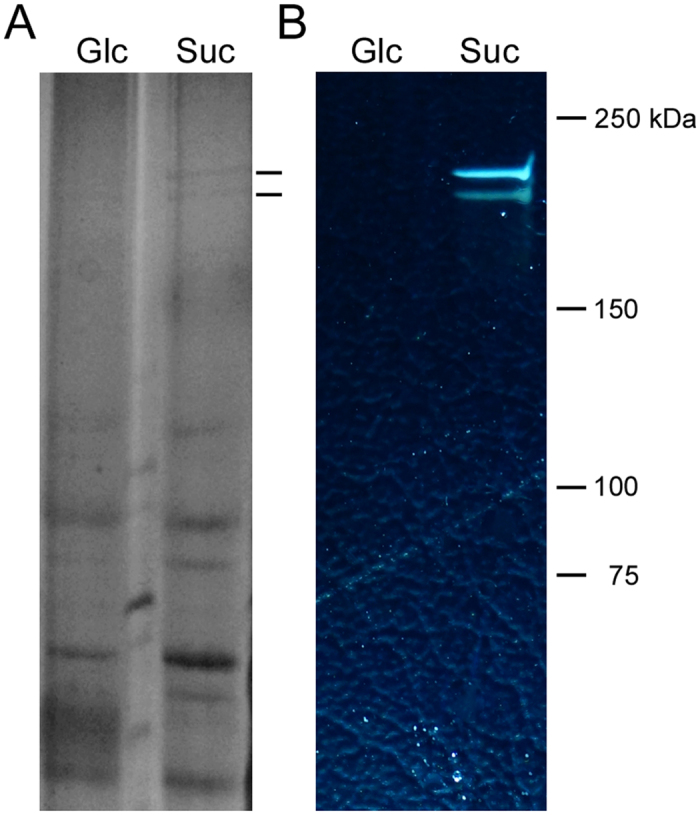
Identification of glucansucrase Gsy in BD3749. (**A**) The total proteins from the supernatant were prepared as described and equivalent amount of total protein was separated on a 16 × 20 cm 6% SDS-PAGE and stained with Coomassie brilliant blue G250. The lines on the right indicate the differentially expressed protein bands. (**B**) *In situ* detection of dextransucrase activity. After electrophoresis, the SDS-PAGE gel was incubated in 50 mM sodium acetate buffer containing 50 g/L sucrose buffered at pH 5.4. The active bands were detected by the appearance of opaque polymer.

A genomic study of BD3749 suggested that there are four putative glucansucrase-encoding genes (*BD3749_1322, 1323, 1645* and *1650*) (Table 2). To identify the glucansucrase responsible for EPS synthesis and cell aggregation, the secreted protein of BD3749 in sucrose medium was analyzed by SDS-PAGE (Fig. 3A). Two proteins with relative molecular weight of ~180 kDa were differentially expressed in sucrose medium, compared to that from glucose medium. To further characterize the differentially expressed proteins, an equivalent gel was subjected to *in situ* polymer synthesis in sucrose-containing buffer. As shown in Fig. 3B, these two differential bands were sucrose-active, catalyzing polymer synthesis from the sole substrate sucrose.

**Figure 4:**
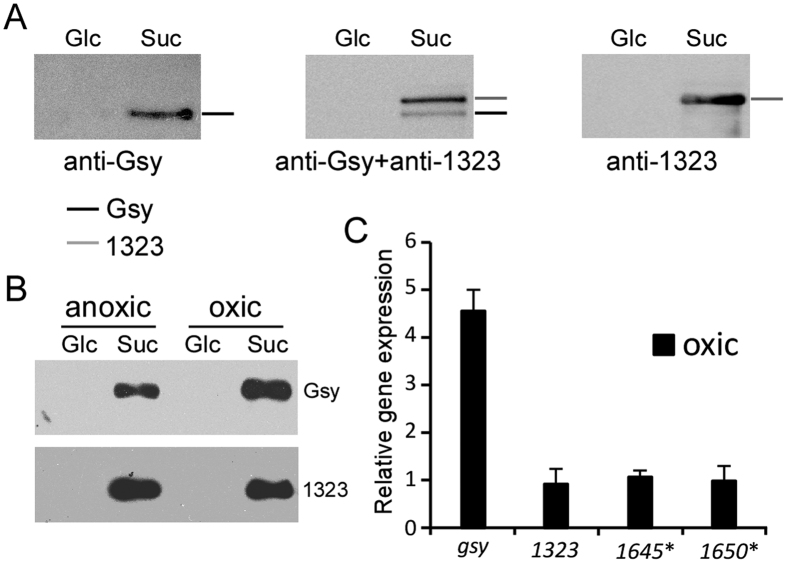
Expression analysis of glucansucrase-encoding genes. (**A**) Equal amount of total protein from the supernant was separated by 6% SDS-PAGE and immunoblotted with the indicated antibodies after transfer onto PVDF membranes. The gel was run in triplicate and analysed with anti-1322, anti-1323, and anti-1322 plus anti-1323, respectively. (**B**) Effect of oxygen on the protein level of glucansucrases. Equal amount of total protein from cell pellets from oxic and anoxic cultures were separated and detected with the indicated antibody. (**C**) Transcription analysis of the putative glucansucrase genes in response to oxygen. The transcription level under oxic conditions was normalized to that under anoxic conditions. Asterisk indicates low transcription level that is close to the limit of detection.

Moreover, immunoblot analysis (Fig. 4A) with antibodies specific to BD3749_1322 (renamed Gsy in this work) and BD3749_1323 suggested that 1322 specific antibody recognized the lower of the two differential bands, while the 1323 specific antibody reacted with the upper one. This pattern coincided with the molecular weights calculation suggesting that (with a calculated molecular weight of 164 kDa) has a smaller molecular weight than 1323 (with a calculated molecular weight of 170 kDa).

### Gsy, a novel glucansucrase in the GH70 family, mediates aggregation

To elucidate the mechanism of oxygen-induced cell aggregation, the expression level of the four glucansucrase-encoding genes in response to oxygen were studied. First, the protein levels of 1322 and 1323 were analysed by western blot analysis. As shown in Fig. 4B, 1322 was up-regulated following exposure to oxygen, whereas 1323 was not affected. Moreover, a transcriptional study of the glucansucrase genes demonstrated that 1322 was induced under oxic conditions, whereas 1323 displayed a relatively constant level of transcription (Fig. 4C), indicating that oxygen-induced aggregation could be mediated by 1322. Interestingly, the other two glucansucrase genes, *BD3749_1645* and *1650* were not induced by sucrose or oxygen.

**Figure 5:**
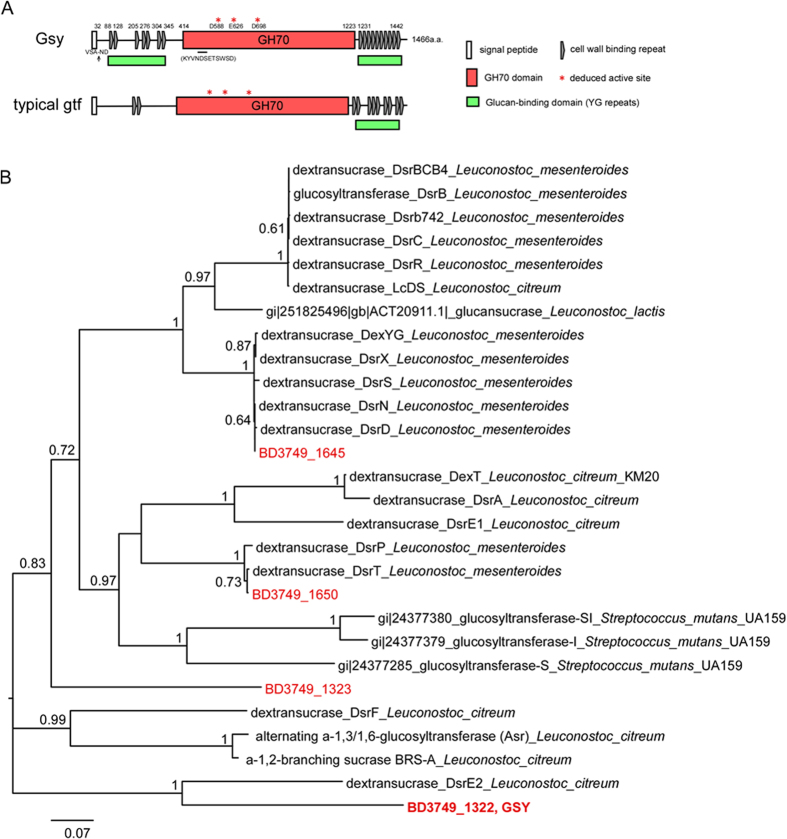
Sequence-based analysis of Gsy. (**A**) Domain structure analysis of Gsy. The deduced active sites are marked with asterisks, and the peptide used for antibody production is indicated below the GH70 domain. Cell wall binding repeats were analyzed on Scanprosite (http://prosite.expasy.org/scanprosite). Shown below Gsy is a typical Gtf from *S.mutans* (AAN58706.1)(http://www.cazy.org/GH70_characterized.html, http://prosite.expasy.org/scanprosite/). (**B**) Phylogenetic analysis of putative glucansucrases in BD3749 and the characterized GH70 enzymes. The tree was constructed with the maximum-likelihood method based on the JTT matrix-based model using MEGA6^57^. Each protein is labelled with its GenBank accession number. GH70 enzymes of BD3749 are labelled in red. The value on each branch is the estimated confidence limit for the position of the branches, as determined by bootstrap analysis. The scale bar represents a 7% difference in amino acid sequence^58^. It should be noted that CDX66820.1 and CAB65910.2, GH70 enzymes from NRRL B-1299 and NRRL B-1355, respectively, were assigned to *Leuconostoc citreum* as suggested by Bounaix *et al*.^59^.

Sequence-based analysis of BD3749_1322 suggested that it is a member of the GH70 family (Glycoside Hydrolase family 70). The 1322 consists of a leading signal peptide at the N-terminus, which is responsible for the extracellular secretion of protein, a glucan-binding domain composed of YG repeats^40^ at both termini and a catalytic GH70 domain localized between these two glucan-binding domains (Fig. 5A). Alignments with other characterized GH70 enzymes demonstrated that Gsy harbours clustered YG repeats at both N- and C-termini of the protein. Thus, 1322 is referred to as Gsy (glucansucrase with YG repeats) in this study to emphasize its rich content of YG repeats. Among the characterized GH70 glucansucrases, Gsy shares the highest similarity with Catalytic Domain 2 (CD2) of *Dsr*E from *L. citreum* NRRL B-1299^41^,^42^ based on a BLAST analysis. The identity between Gsy and CD2-*Dsr*E is 50%. Moreover, a phylogenetic analysis demonstrated that Gsy shared relatively low similarity with most of the characterized GH70 enzymes (Fig. 5B).

**Figure 6:**
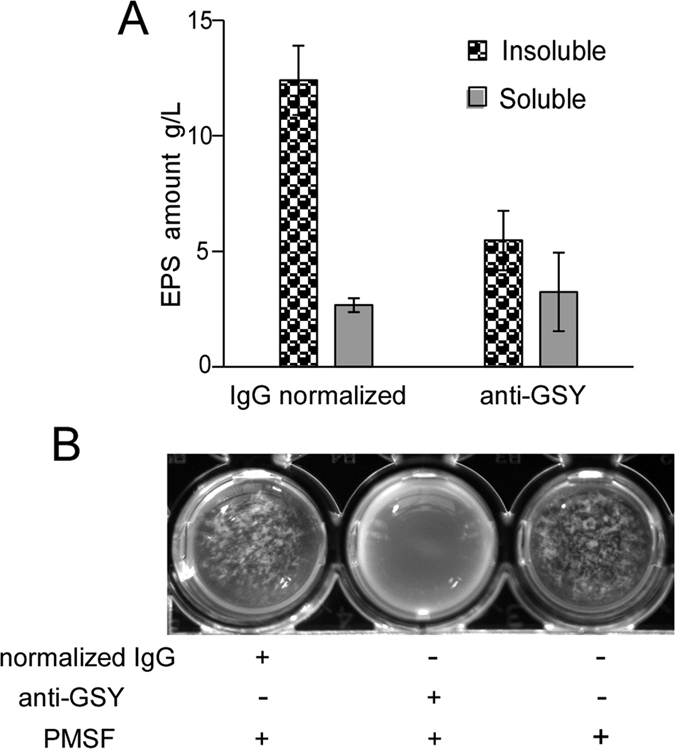
Functional blockade of Gsy inhibits aggregation. (**A**) The Gsy antibody inhibited the production of insoluble EPS. The antibody was supplemented as mentioned above, and EPS were collected as described in Materials and Methods. The average amount from three experiments and standard deviations are shown. (**B**) Gsy antibody blocked oxygen-induced aggregate formation. Antibody was added to a final concentration 20 μg/mL, and normalized IgG was used as a negative control. PMSF (100 μM) was added to prevent antibody degradation. The primitive culture was photographed to demonstrate the formation of aggregates.

The role of glucansucrases in EPS synthesis and cell aggregation was also investigated. In a function blockade assay performed as previously described by Chia *et al*.^43^,^44^, a Gsy-specific antibody effectively inhibited the synthesis of insoluble EPS (Fig. 6A) as well as cell aggregation (Fig. 6B), while a 1323-specific antibody showed no obvious effect on either process, suggesting that 1322 is responsible for the synthesis of insoluble EPS and plays an important role in the formation of cell aggregates.

### Oxidative stress stimulates the synthesis of insoluble EPS

**Figure 7:**
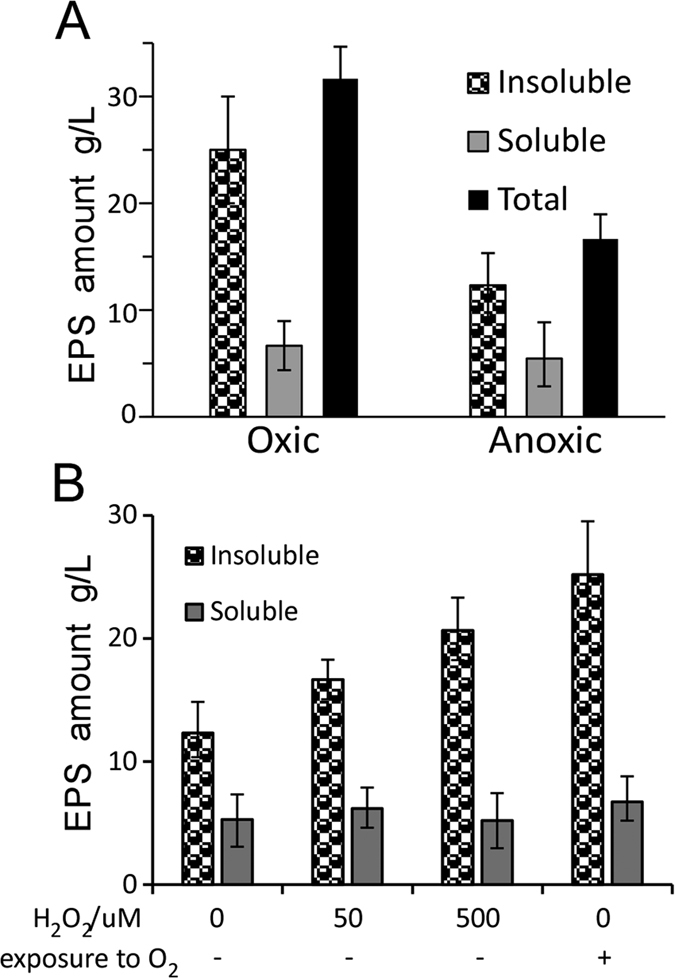
Increase in EPS in response to oxidative stress. (**A**) EPS synthesized by BD3749 under oxic and anoxic conditions. The insoluble and soluble EPS were collected and detected, respectively. (**B**) EPS synthesized by BD3749 in broth with H_2_O_2_ log phase culture of BD3749 was diluted 1:50 into fresh medium supplemented with H_2_O_2_ at the indicated concentration. After 48 h of cultivation, insoluble and soluble EPS in the culture were obtained as described in Materials and Methods. Excluding the oxic group, incubation was carried out in the Whitley A35 Anaerobic Workstation. The average amount of three experiments and standard deviations are shown.

Because the insoluble EPS synthesized by BD 3749 cells under oxidative stress made up the majority of the aggregates, it could be speculated that additional insoluble EPS would be produced as the intensity of the oxidative stress increased. To testify this speculation, the EPS amount of BD3749 in an optimized medium containing 200 g/L sucrose were quantified as described by Liu *et al*.^31^. As shown in Fig. 7A, the amount of insoluble EPS was 25 g/L under oxic conditions, but under anoxic conditions, it was 12.3 g/L. It could thus be easily deduced that the synthesis of insoluble EPS was greatly stimulated when BD3749 cells were exposed to oxidative stress. The increase in the amount of insoluble EPS in response to oxidative stress could be explained by the up-regulation of Gsy upon exposure to oxygen (Fig. 4B and C). Interestingly, exposure to oxygen only slightly increased the amount of soluble EPS (Fig. 7A), indicating that the synthesis of insoluble and soluble EPS were performed by distinct glucansucrases that might be regulated by different mechanisms.

Since exposure to oxygen could stimulate BD3749 cells to synthesize EPS, H_2_O_2_ was selected as another source of oxidative stress to test whether it could exert a similar impact on the bacterial cells. Like most other strains of *L. mesenteroides*, BD3749 displayed a high sensitivity to H_2_O_2._ At 50 μM and 500 μM, treatment with H_2_O_2_ could significantly promote BD3749 cells to synthesize insoluble EPS (Fig. 7B). Therefore, oxidative stress would greatly stimulate BD3749 to synthesize EPS, especially insoluble ones, which would subsequently mediate the formation of aggregates to protect the bacterial cells.

### EPS improve the survival of BD3749 under chronic oxygen stress

**Figure 8:**
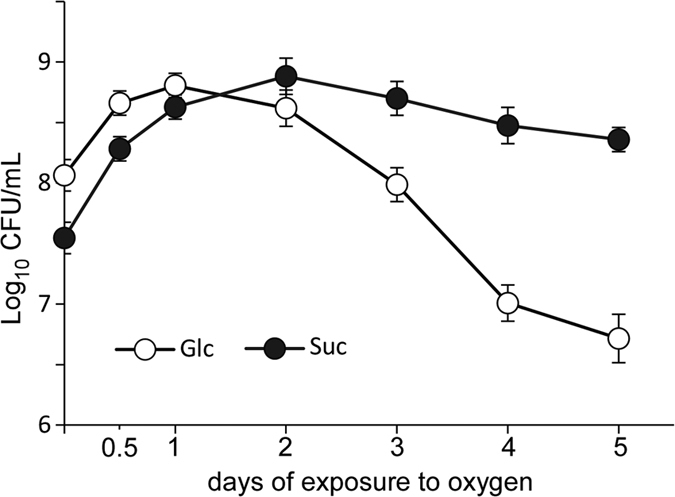
Exopolysaccharides improve the survival of BD3749 cells under chronic oxygen stress. BD3749 cells were inoculated into 30 mL TYC-Suc medium or TYC-Glc medium (in which sucrose was substituted with an equal amount of glucose) and incubated anaerobically at 37 °C for 2 days. The cultures were then shifted to an aerobic incubator at 30 °C and maintained for 5 days, during which the pH value was monitored and viable cells were enumerated by plate counting. The average amount of three experiments and standard deviations are shown in the graph.

As mentioned above, EPS synthesized by BD3749 in medium containing sucrose as the carbon source could reduce the accumulation of ROS in the bacterial cells and thus alleviate the challenge of oxidative stress to the cells. Thus, EPS would improve the survival of BD3749 cells under chronic oxygen stress. As shown in Fig. 8, although viable cell counts in TYC medium containing 50 g/L of either sucrose or glucose in the first 24 h of cultivation differed insignificantly, the survival rate of BD3749 cells in the sucrose medium were much higher than those in the glucose medium afterwards. After 5 days exposure to oxygen, viable cells in glucose medium were less than 10^7^ CFU/mL, while those in sucrose medium were 5 × 10^8^ CFU/mL. As no other difference were observed between the two cultures, excluding the EPS contents, the higher survival ratio of BD3749 cells in sucrose medium could be attributed to the protection of EPS. A similar phenomenon was observed in a recent study^45^, in which EPS produced in sucrose exhibited important improvement of the survival of the gut commensal *Lactobacillus reuteri*. Therefore, EPS produced in sucrose medium could provide BD3749 cells with an efficient protective barrier against oxidative stress caused by chronic exposure to oxygen.

## Discussion

Glucansucrase-encoding genes, *e.g. dsr* genes have been found in most *L. mesenteroides* strains. Typically, these genes account for ~1% of the total genomic DNA. Full-length genomic DNA sequencing demonstrated that four glucansucrase-encoding genes are present in *L. mesenteroides* BD3749, accounting for 1.1% of the genomic DNA. Additionally, BD3749 produced large amount of EPS when cultivated in sucrose medium. These characteristics suggested that glucansucrases, and their enzymatic product EPSs, might play important roles in the growth and survival of BD3749 cells in both aerobic environments. EPS, including insoluble ones, have been widely studied in LABs such as *Streptococcus mutans* and *Lactobacillus reuteri*, and their role in biofilm formation has been elucidated. In this study, the role of insoluble EPS in the aggregation of *L. mesenteroides* was assessed, and their biological significance in response to oxidative stress was addressed.

In response to the oxidative stress caused by environmental oxygen, *L. mesenteroides* BD3749 adopted a strategy in which most of bacterial cells formed aggregates to escape the oxygen challenge, through the up-regulation of the glucansucrase-encoding gene *gsy*. The results regarding up-regulation of Gsy and enhanced synthesis of EPS when exposed to oxygen are consistent with previous studies, in which oxidative stress could promote the individual bacterium to synthesize EPS^46^,^47^,^48^. Cell aggregation is complicated process that may involve cell wall-associated proteins, glucan-binding proteins, and extracellular substances other than EPS, such as extracellular DNA^49^,^50^. In this study we showed that glucansucrase Gsy played an important role in cell aggregation, however, other secreted proteins and cell wall proteins, such as glucan-binding proteins, may also be involved, thus studies will be needed to fully identify molecules involved in this process in the future.

In addition to the role of aggregation discussed above, other mechanisms might also be present in catalase-negative bacteria to escape from oxidative stress. Some anaerobic bacteria have been shown to form biofilms to generate a hypoxic microenvironment for the presence of the bacterial cells^51^ or to take advantage of fungal biofilms to reduce the detrimental effect of oxygen for their growth^52^. For BD3749, soluble EPS secreted into the medium can extrude dissolved oxygen, which appeared to act as a backup barrier against oxygen (Fig. S2).

Gsy, which was induced by the presence of sucrose in the surroundings, was up-regulated in response to oxidative stress in BD3749 cells, the enzymatic product of which, i.e., insoluble EPS, mediated the formation of cell aggregates. Gsy represented a category of uncharacterized glucansucrase in *L. mesenteroides* in the GH70 family based on a sequence analysis because the similarity between Gsy and the characterized GH70 enzymes was relatively low. Interestingly, among the characterized GH70 enzymes, Gsy was close to the α-1,2-branching sucrase, CD2-*Dsr*E^42^, and the newly identified BRS-A^53^ of *L. citreum* NRRL B-1299 in the phylogenetic analysis. However, when aligned with the identified branching sucrases, in the conserved motifs, Gsy displayed a distinct pattern at sites that were exclusively conserved in the branching sucrases (Fig. S3). Moreover, an *in situ* polymer synthesis assay demonstrated that Gsy can produce polymer *de novo* from sucrase alone (Fig. 3). Additionally, heterologously expressed Gsy catalyzed the synthesis of polymer from sucrose (Fig. S4). Thus, Gsy is a glucansucrase that synthesizes EPS from the substrate sucrose.

The strategy employed by BD3749 cells to avoid oxidative stress does not appear to be a special case as insoluble EPS has been reported previously in *L. mesenteroides*^54^, and EPS-mediated aggregation appears to occur frequently in nature. For example, the aggregated granules in activated sludge, composed of facultative and anaerobic bacteria in the core, surrounded by microorganisms that are less sensitive to oxygen are widely applied for the treatment of wastewater^38^,^55^. During the formation of granules, the content of EPS (extracellular polymeric substances, most of which are polysaccharides) would increase with an increasing size of the granules^38^. Recently, it has been reported that in aggregates composed of N_2_-fixing cyanobacterial and other non-oxygen-releasing bacteria, there are anoxic microniches within the aggregates even when they were suspended in oxygen-saturated waters^56^. These findings could provide partial support for our conclusion that EPS-mediated aggregation plays critical roles in the escape of anaerobes from oxidative stress caused by environmental oxygen. Interestingly, a considerable amount of insoluble EPS was synthesized under anoxic conditions, although to a much lower extent than that under oxic conditions by BD3749 with available sucrose (Fig. 7A). However, no obvious aggregation could be observed under anoxic conditions, indicating that other oxygen-induced protein (s) may be involved in aggregation. Further studies are needed to fully address the molecular details responsible for aggregation.

## Additional Information

**How to cite this article**: Yan, M. *et al*. Gsy, a novel glucansucrase from *Leuconostoc mesenteroides*, mediates the formation of cell aggregates in response to oxidative stress. *Sci. Rep.* **6**, 38122; doi: 10.1038/srep38122 (2016).

**Publisher's note:** Springer Nature remains neutral with regard to jurisdictional claims in published maps and institutional affiliations.

## Supplementary Material

Supplementary Information

## Figures and Tables

**Table 1:** Specific primers used for real-time PCR.

Primers	Sequences
16 S rRNA	5′-AGCGTTATCCGGATTTATTG -3′
	5′-CTACGCATTCCACCGCTACA-3′
*gsy*	5′-GGATGCTGTTATATATATGA-3′
	5′-ACTGGCTATCTTGGCAAGTG-3′
*BD3749_1323*	5′-GACTTGCTACAAATTGCTGC-3′
	5′-CGATCGACATTTCTATTGTTA-3′
*BD3749_1645*	5′-GAAGATTGGAGTCACAATGA-3′
	5′-GTTTGCACTTCGCTGTCGTG-3′
*BD3749_1650*	5′-ATACCGCACAACCAAATTAT-3′
	5′-ACACACGTGGCACTGTATCT-3′

**Table 2:** Putative GH70 family glucansucrase deduced from the genome sequence of BD3749^a^.

Putative GH70s	*dsr* genes	length	description	Accession No.
GH70-1	*BD3749_1322*	1466a.a.	No homolog with identity of more than 50% was reported, identified as Gsy in this manuscript.	KU306931
GH70-2	*BD3749_1323*	1513a.a.	97% Identity with AHF19405.1, Glycosyl hydrolase family 70 with YG repeats (*L. mesenteroides* KFRI-MG).	KU306932
GH70-3	*BD3749_1645*	1527a.a.	Homolog of DsrD (dextransucrase of *L. mesenteroides* Lcc4), ID = 99%	KU306933
GH70-4	*BD3749_1650*	1479a.a.	Homolog of DsrP (cell-associated dextransucrase produced by *L. mesenteroides* IBT-PQ), ID = 93%	KU306934

^a^The genome sequence of BD3749 has been submitted to GenBank under accession number CP014610.1.
