# Supplementary material for: Gsy, a novel glucansucrase from Leuconostoc mesenteroides, mediates the formation of cell aggregates in response to oxidative stress
Source: Sci Rep. 2016 Dec 7;6:38122. doi: 10.1038/srep38122 (PMC5141493; doi:10.1038/srep38122)
Supplement: Supplementary Information [file srep38122-s1.pdf]

GSY, a novel glucansucrase from *Leuconostoc mesenteroides*, mediates the formation of cell aggregates in response to oxidative stress

Minghui Yan, Jin Han, Xiaofen Xu, Lianliang Liu, Caixia Gao, Huajun Zheng, Yunxia Chen, Yimin Tao, Hu Zhou, Yunfei Li and Zhengjun Wu

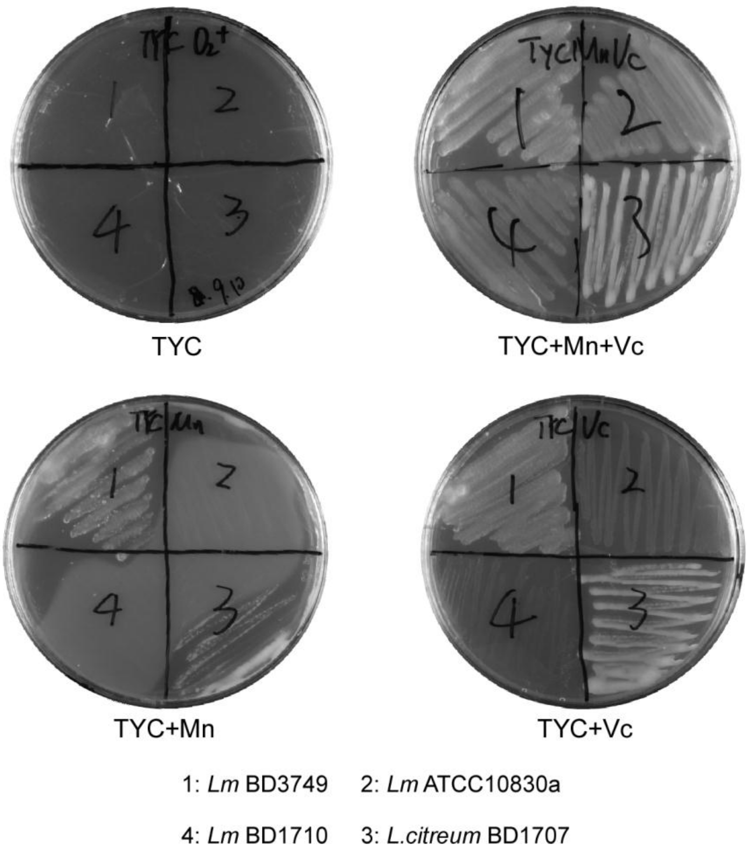

Fig.S1. Antioxidants (Mn and Vc) dependent growth of BD3749 exposed to oxygen

2uL of log phase culture of stains of *Leuconostoc* (*L. mesenteroides* BD3749, ATCC10830a, BD1710 and *L.citreum* BD1707) were spotted and streaked onto TYC plates with or without the indicated antioxidants ($MnSO_4$: 0.25g/L[1], Vitamin C: 0.5g/L[2]). The plates were then incubated aerobically at 30°C. The photos were taken after 3 days of growth.

ATCC10830a was bought from ATCC, BD1710[3] and BD1707[4] was provided by State Key Laboratory of Dairy Biotechnology.

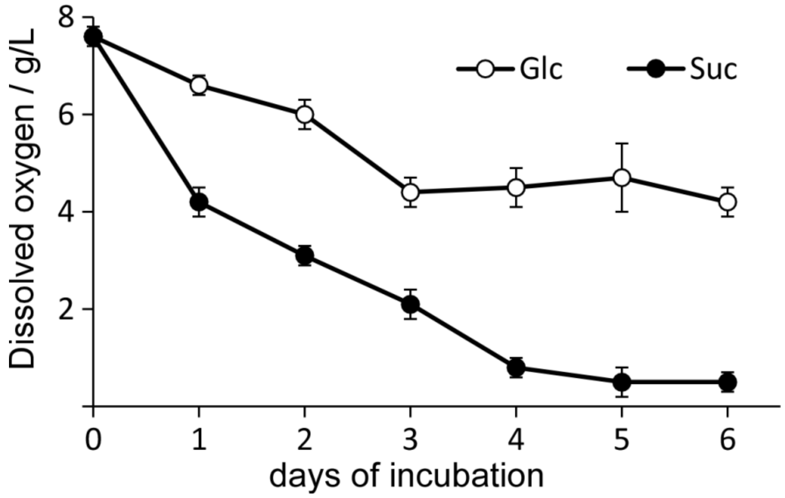

Fig.S2. Soluble EPS promotes exclusion of oxygen from the culture medium

Overnight culture of BD3749 was 1:100 diluted in triplicate into fresh TYC media with glucose (Glc) or sucrose (Suc) as the sole carbon source and incubated aerobically at 30° C. Dissolved oxygen was detected every 24 hours with JPB-607A portable Dissolved Oxygen Meters (INESA Scientific Instrument Co., Ltd). Experiments were performed in triplicate, and the average concentration of Dissolved oxygen are shown with error bars indicate the standard deviations.

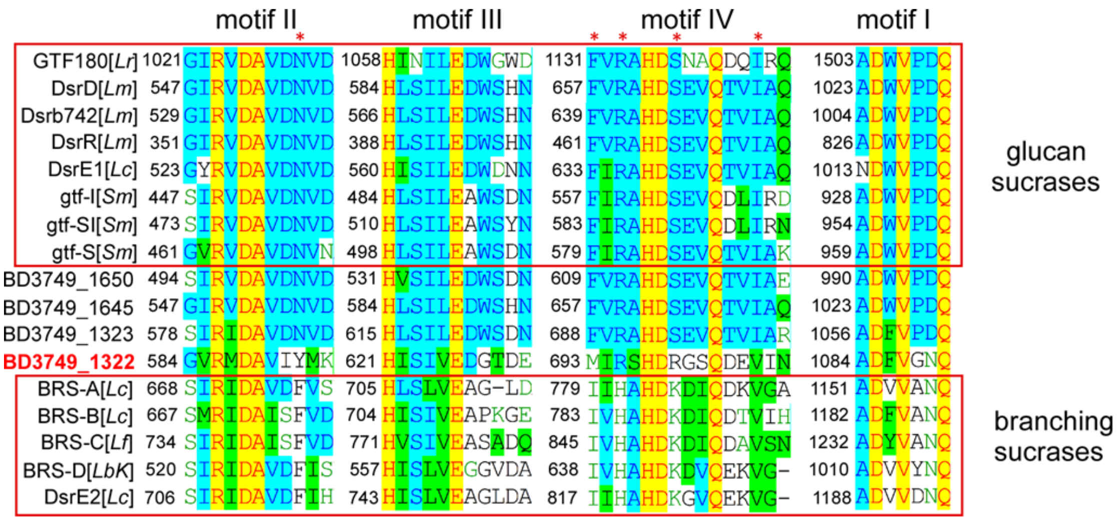

Fig.S3. Sequence alignment of the conserved motifs (I-IV) in catalytic core of branching sucrases with that of glucansucrases in Leuconostocs

The sequence of conserved motifs (I-IV) of glucansucrases and branching sucrases were aligned with Align X (Invitrogen). The amino acids that are only conserved in branching sucrases (F675, I783, H785, K789 and V795 for BRS-B) [5]are marked with asterisks. Gsy (BD3749_1322) shows a pattern different from either the glucansucrases or branching sucrase at these sites, while the other three shows a same pattern with glucansucrases.

[*Sm*], *Streptococcus mutans*, [*Lr*], *Lactobacillus reuteri*, [*Lm*], *Leuconostoc mesenteroides*, [*Lc*], *Leuconostoc citreum*, [*Lf*], *Leuconostoc fallax*, [*LbK*], *Lactobacillus kunkeei*

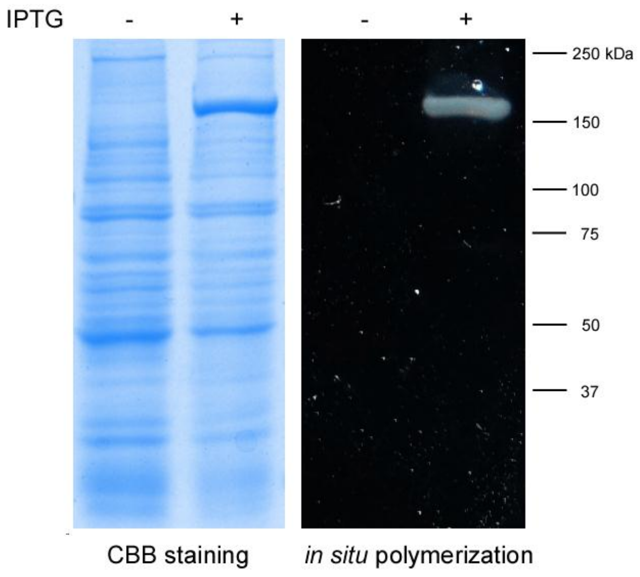

Fig.S4. Polymerization activity of heterologously expressed Gsy

gsy gene was PCR amplified from genomic DNA of BD3749 and integrated into the vector pET-28a. The pET28a-*gsy* plasmid was then transformed in Rosetta strains of E. coli as previously described[6]. Isopropy-β-D-thiogalactoside (IPTG) was added into the culture medium to a final concentration of 0.1mM for induction of the recombinant Gsy. Crude extract of *E.coli* was prepared and SDS-PAGE was performed as described previously[6,7] and *in situ* polymerization was carried out as described in materials and methods.

1. Cheng, X., Dong, Y., Su, P. & Xiao, X. Improvement of the Fermentative Activity of Lactic Acid Bacteria Starter Culture by the Addition of Mn2+. *Appl Biochem Biotechn* **174**, 1752-1760 (2014).
2. Porro, D., Branduardi, P., Mattanovich, D., Sauer, M. Increase in stress tolerance with ascorbic acid during fermentation. *United States Patent Application 20070141687*, A1 (2007).
3. Han, J. *et al.* Dextran synthesized by Leuconostoc mesenteroides BD1710 in tomato juice supplemented with sucrose. *Carbohydr Polym* **112**, 556-562 (2014).
4. Han, J., Xu, X., Gao, C., Liu, Z. & Wu, Z. Isolation of a levan producing Leuconostoc citreum strain BD1707 and its growth characterization in tomato juice supplemented with sucrose. *Appl Environ Microbiology* **82**(5), 02944-02915 (2015).
5. M, V. *et al.* Characterization of the First α-(1→3) Branching Sucrases of the GH70 Family. *J Biol Chem* **291**(14) (2016).
6. Ruhmkorf, C. *et al.* Identification of Lactobacillus curvatus TMW 1.624 dextransucrase and comparative characterization with Lactobacillus reuteri TMW 1.106 and Lactobacillus animalis TMW 1.971 dextransucrases. *Food Microbiol* **34**, 52-61, doi:10.1016/j.fm.2012.11.002 (2013).
7. Hanada, N. & Kuramitsu, H. K. Isolation and characterization of the Streptococcus mutans gtfC gene, coding for synthesis of both soluble and insoluble glucans. *Infect Immun* **56**, 1999-2005 (1988).
